# Association Between Early Childhood Caries and Obesity among Preschool Children

**DOI:** 10.3290/j.ohpd.b2805445

**Published:** 2022-03-14

**Authors:** Roshan Noor Mohamed, Sakeenabi Basha, Yousef Al-Thomali, Fatma Salem AlZahrani, Amal Adnan Ashour, Nada Eid Almutair

**Affiliations:** a Assistant Professor, Department of Pedodontics, Faculty of Dentistry, Taif University Taif, Saudi Arabia. Conception and design; data acquisition, analysis, and interpretation; drafted and critically revised manuscript, gave final approval; agreed to be accountable for all aspects of the work.; b Assistant Professor, Department of Community Dentistry, Faculty of Dentistry, Taif University Taif, Saudi Arabia. Conception and design; data acquisition, analysis, and interpretation; drafted and critically revised manuscript, gave final approval; agreed to be accountable for all aspects of the work.; c Associate Professor, Department of Orthodontics, Faculty of Dentistry, Taif University Taif, Saudi Arabia. Conception and design; data acquisition, analysis, and interpretation; drafted and critically revised manuscript; agreed to be accountable for all aspects of the work.; d Assistant Professor, Department of Pedodontics, Faculty of Dentistry, Taif University Taif, Saudi Arabia. Conception and design; data acquisition, analysis, and interpretation; drafted and critically revised manuscript; agreed to be accountable for all aspects of the work.; e Assistant Professor, Department of Oral Pathology, Faculty of Dentistry, Taif University Taif, Saudi Arabia. Conception and design; data acquisition, analysis, and interpretation; drafted and critically revised manuscript; agreed to be accountable for all aspects of the work.; f Community Services Coordinator, Department of Community Dentistry, Faculty of Dentistry, Taif University, Taif, Saudi Arabia. Conception and design; data acquisition, analysis, and interpretation; drafted and critically revised manuscript; agreed to be accountable for all aspects of the work.

**Keywords:** ECC, obesity, preschool, prevalence

## Abstract

**Purpose:**

Early childhood caries (ECC) and childhood obesity are among the most prevalent health conditions affecting children. ECC is associated with obesity through the common risk factor of sugar consumption. The present study aimed to assess the association between ECC and obesity in preschool children.

**Materials and Methods:**

A cross-sectional study was conducted among 1250 preschool children (698 girls, 552 boys; mean age: 4.3 [1.1] years). The children’s body mass index was determined (BMI: weight/height in kg/m^2^). The World Health Organization criteria were used for the diagnosis of caries. Multivariable logistic regression was used to analyse the relationship between ECC prevalence and childhood obesity.

**Results:**

ECC was detected in 929 (74.3%) children. The mean dmft and dmfs was 5.91 (1.13) and 8.92 (2.07), respectively. The multiple regression model showed a statistically significant association between ECC prevalence and obesity with an adjusted odds ratio (OR) of 2.59 (95% CI: 1.88 – 3.57; P = 0.001). The logistic regression model showed that in children with a monthly family income > $2666, sugar consumption, preterm low birth-weight/full-term low birth-weight (PTLBW/FTLBW), and toothbrushing frequency ≤ 1 time/day were statistically significantly associated with ECC prevalence.

**Conclusion:**

ECC was positively associated with obesity.

Early childhood caries (ECC) and childhood obesity are among the most prevalent health conditions affecting children.^28^ ECC affects children early in life and progresses rapidly, potentially leading to tooth mortality.^8^ Untreated ECC affects the child’s quality of life and also presents a financial burden on parents.^1,19^ According to a recent systematic review, the global prevalence of ECC was 48% (95% CI: 42-53).^30^ The previous point prevalence studies conducted in Saudi Arabia showed a high prevalence of ECC (72% to 77%) among preschool children.^3,4^ ECC is a multifactorial condition influenced by several risk factors, such as high sucrose diet, poor oral hygiene, presence of enamel defects, oral microbial flora, prolonged breastfeeding/bottle feeding, and sociodemographic factors.^6,16,18,33^

Childhood obesity is another highly prevalent global health problem; as many as 40 million children under the age of 5 years were overweight or obese, according to a global nutrion report.^11^ Obesity increases the risk of non-communicable diseases, e.g. diabetes, cardiovascular diseases, and musculoskeletal problems.^14,21,27^ Along with these general health risks, obesity is associated with the most common oral disease among children: dental caries.^20^ A high-sugar diet is a common risk factor associated with both conditions.^20,23^ Previous published research showed inconsistent results, with a few studies showing positive association,^5,10,12,31^ a few with a negative association,^6,22,24^ and one with no association between ECC and obesity.^9^ A recent systematic review showed an equivocal association between the two conditions and highlighted the need for further research in this regard.^20^ The results of previous studies were inconclusive; consistency in establishing an association between obesity and ECC was lacking.^6,5,9,10,12,22,24,31^ Hence, the present study aimed to investigate the association between obesity and ECC among preschool children in Taif City, Saudi Arabia.

## Materials and Methods

### Study Population, Study Design, And Sample Size

A cross-sectional study was conducted among 1250 preschool children (698 girls, 552 boys; mean age: 4.3 [1.1] years) in Taif City, Makkah Province, KSA. Based on the pilot study (n = 50), a sample size of 1250 was chosen (population proportion of 0.35, power of 80%, type 1 error 0.5%). Thirty public schools were selected by randomised lottery number from five city zones (five to seven schools from each city zone). The required sample size of preschool children was selected by probability proportional to the size random sampling technique (30 to 50 preschool children from each school). The Institutional Review Board approved the study (IRB approval number: 39-11007-0029). The parents/guardians permitted the participation of their children/wards through written informed consent. Children under 71 months old were included, in accordance with the ECC definition criteria.^30^

### Questionnaire

The relevant information was collected from parents/guardians using pretested structured questionnaire. The internal consistency was checked by distributing the questionnaire to 25 guardians/parents. Cronbach’s alpha was 0.95. The sociodemographic details (age in years, gender, family income, years of mother’s education), and dietary habits (72-h diet recording including 2 weekdays and a weekend). The frequency, form, time of sugar intake (with or between meals), as well as the sugar’s consistency (sticky or not) were recorded, as were history of bottle feeding at night with sugared drinks, preterm or full-term birth, birth weight, oral hygiene practices (method, material used, frequency, and tooth-cleaning time [morning or evening]). The family income categorisation was based on the Household Income and Expenditure Survey, Saudi Arabia, 2018.^15^

### Anthropometric Measurements

The criteria described by Al-Herbish et al^2^ were utilised to categorise the children according to BMI into 4 groups based on their age and gender. Underweight: < 5th percentile; normal weight: 5th percentile to < 84th percentile; overweight: 85th < 95th percentile; obese: ≥ 95th percentile.

### Oral Examination

A single examiner examined all the preschool children under natural light using sterile mouth mirrors and CPI probes. Caries diagnosis (dmft or dmfs) was done according to the WHO criteria.^32^ Occlusal and cavitated proximal lesions were recorded using the visual and tactile method without radiographs. 15% of participants were examined twice in succession to determine intra-examiner consistency for caries diagnostic criteria (Kappa value of 0.92, p < 0.05).

### Statistical Analysis

The difference in proportion of ECC presence/absence and BMI categories in relation to the variables studied was tested using chi-squared tests, followed by the pair-wise Z-test with Bonferroni’s correction for intergroup comparison. The mean difference was tested using Student’s t-test and one-way ANOVA, followed by Tukey’s post-hoc analysis for intergroup comparison. Multiple logistic regression analysis was used to determine the relationships between ECC prevalence (yes/no) and obesity, controlling for the covariates. Logistic stepwise regression analysis with backward elimination and forward entry was used to assess the impact of all the covariates on ECC prevalence (yes/no). Covariates included gender, diet, oral hygiene practice, preterm or full-term low birth weight, years of mother’s education, and family income. IBM SPSS Statistics program version 22 (IBM; Armonk, NY, USA) was used for statistical analysis. All statistical tests were two-sided, and the significance level was set at p < 0.05.

## Results

Of the 1250 children, 227 (18.2%) were overweight, and 175 (14%) were obese. The mean BMI for the whole study population was 21.8 (2.7). Table 1 present the BMI categories according to gender, family income, and dietary factors. No statistically significant difference was observed between BMI categories and the studied variables. The overall prevalence of ECC was 74.3%. Severe ECC was recorded among 357 (28.6%) children. Children of families with a monthly income > $2666 had a statistically significantly higher prevalence (84%) of ECC (P = 0.001). Obese (88.6%) and overweight children (81.1%) had a statistically significantly higher prevalence of ECC (P = 0.001) (Table 2). ECC was present in 929 children, with mean dmft and dmfs of 5.91 (1.13) and 8.92 (2.07), respectively (Table 3).

**Table 1:** BMI categories according to gender, diet, and family income

Variable	BMI categories, n (%)				Chi-squared test, p-value
	Underweight	Normal weight	Overweight	Obese	
**Gender**					0.153
Boys (n = 552)	12 (2.2)a	367 (66.5)a	87 (15.8)a	86 (15.6)a	
Girls (n = 698)	18 (2.6)a	451 (64.6)a	140 (20.1)a	89 (12.8)a	
**Sugar consumption**					0.254
Yes (n = 838)	17 (2.0)a	542 (64.7)a	163 (19.5)a	116 (13.8)a	
No (n = 412)	13 (3.2)a	276 (67.0)a	64 (15.5)a	59 (14.3)a	
**Monthly family income**					0.056
≤ $2666 (n = 913)	18 (2.0)a	608 (66.6)a	171 (18.7)a	116 (12.7)a	
> $2667 (n = 337)	12 (3.6)a	210 (62.3)a	56 (16.6)a	59 (17.5)a	
**Bottle feeding at night with sugared drinks**					0.116
Yes (n = 725)	14 (1.9)a	490 (67.6)a	131 (18.1)a	90 (12.4)a	
No (n = 525)	16 (3.0)a	328 (62.5)a	96 (18.3)a	85 (16.2)a	

a: Z-test with pair-wise comparison using Bonferroni correction, p > 0.05; $: US dollars.

**Table 2:** Early childhood caries (ECC) prevalence according to variables studied

Variables	ECC		Chi-square test, p-value
	Present, n (%)	Absent, n (%)	
**Gender**			0.072
Boys (n = 552)	439 (79.5)	113 (20.5)	
Girls (n = 698)	490 (70.2)	208 (29.8)	
**Monthly family income**			0.001
≤ $2666 (n = 913)	646 (70.8)	267 (29.2)	
> $2666 (n = 337)	283 (840.0)	54 (160.0)	
**Mother’s education**			0.604
0 years of education (n = 299)	221 (73.9)a	78 (26.1)a	
Primary school (n = 466)	356 (76.4)a	110 (23.6)a	
Secondary school (n = 309)	224 (72.5)a	85 (27.5)a	
High school and university (n = 176)	128 (72.7)a	48 (27.3)a	
**Sugar consumption**			0.083
Yes (n = 838)	640 (76.4)	198 (23.6)	
No (n = 412)	289 (70.1)	123 (29.9)	
**Bottle feeding at night with sugar drinks**			0.286
Yes (n = 725)	534 (73.7)	191 (26.3)	
No (n = 525)	395 (75.2)	130 (24.8)	
**BMI**			0.001
Underweight (n = 30)	19 (63.3)a	11 (36.7)a	
Normal weight (n = 818)	571 (69.8)a	247 (30.2)a	
Overweight (n = 227)	184 (81.1)b	43 (18.9)a	
Obese (n = 175)	155 (88.6)b	20 (11.4)a	
**PTLBW/FTLBW**			0.008
Yes (n = 167)	137 (820.0)	30 (180.0)	
No (n = 1083)	792 (73.1)	291 (26.9)	
**Oral hygiene practices**			
Toothbrushing frequency			0.040
≤ 1 times/day (n = 747)	569 (76.2)	178 (23.8)	
≥ 2 times/day (n = 503)	360 (71.6)	143 (28.4)	
**Use of fluoridated toothpaste**			0.080
Yes (n = 662)	468 (70.7)	194 (29.3)	
No and don’t know (n = 588)	461 (78.4)	127 (21.6)	

ECC: early childhood caries; BMI: body mass index; PTLBW/FTLBW: pre-term low birth-weight/full-term low birth-weight; $: US dollars. a: Z-test with pair-wise comparison using Bonferroni correction, p>0.05; b: p < 0.05.

**Table 3:** Mean caries score (dmft/dmfs) according to gender

Variable	Mean (SD) caries score			
	dt	dmft	ds	dmfs
Girls (n = 698)	5.89 (1.17)	5.95 (1.28)	8.36 (2.14)	9.12 (2.12)
Boys (n = 552)	5.42 (1.12)	5.73 (1.22)	7.18 (1.19)	8.19 (2.03)
t-test, p-value	0.08	0.07	0.053	0.057

dt: decayed tooth; dmft: decayed, missing, filled teeth; ds: decayed surface; dmfs: decayed, missing, filled surface.

The multiple regression model showed a statistically significant association between ECC prevalence and obesity with adjusted odds ratio (OR) of 2.59 (95% CI: 1.88 – 3.57, p = 0.001) (Table 4).

**Table 4:** Multiple logistic regression model showing association between obesity and ECC

Variable	ECC n	ECC %	Unadjusted OR (CI)	Adjusted OR (CI)+
BMI				
Underweight and normal weight (n = 848) #	590	69.6		
Overweight and obese (n = 402)	339	84.3	2.45 (1.73 – 3.43) **	2.59 (1.88 – 3.57) **

#: reference: **p < 0.001; ECC: early childhood caries; CI: confidence interval; OR: odds ratio; +adjusted for gender, family income, mother’s education, preterm birth and low birth weight, sugar diet, bottle feeding at night with sugared drinks, and oral hygiene practices.

The logistic regression model showed that children of families with an income > $2666 had an OR of 2.04 (95% CI: 1.46 – 2.86, p = 0.014). Children with sugar consumption had an OR of 2.02 (95% CI: 1.07 – 3.88, p = 0.015). Children with preterm low birth weight/full term low birth weight (PTLBW/FTLBW) had an OR of 2.15 (95% CI: 1.06 – 4.57, p = 0.024). Children who brushed their teeth ≤ 1 time/day had an OR of 5.63 (95% CI: 3.36 – 9.41, p = 0.001) (Table 5).

**Table 5:** Logistic regression model showing impact of covariates on ECC

Variable	ECC n	ECC %	OR (CI)	p-value
**Gender**				
Girls (n = 698)	490	70.2	Reference	
Boys (n = 552)	439	79.5	0.18 (0.09 – 0.34)	0.342
**Monthly family income**				
≤ $2666 (n = 913)	646	70.8	Reference	
> $2666 (n = 337)	283	84.0	2.04 (1.46 – 2.86)	0.014
**Mother’s education**				
0 to 5 years (n = 765)	577	75.4	0.96 (0.84 – 1.11)	0.600
> 5 years (n = 485)	352	72.6	Reference	
**Sugar consumption**				
Yes (n = 838)	640	76.4	2.02 (1.07 – 3.88)	0.015
No (n = 412)	289	70.1	Reference	
**Bottle feeding at night with sugared drinks**				
Yes (n = 725)	534	73.7	1.10 (0.84 – 1.45)	0.462
No (n = 525)	395	75.2	Reference	
**PTLBW/FTLBW**				
Yes (n = 167)	137	82.0	2.15 (1.06 – 4.57)	0.024
No (n = 1083)	792	73.1	Reference	
**Oral hygiene practices**				
Toothbrushing frequency				
≤ 1 times/day (n = 747)	569	76.2	5.63 (3.36 – 9.41)	0.001
≥ 2 times/day (n = 503)	360	71.6	Reference	
**Use of fluoridated toothpaste**				
Yes (n = 662)	468	70.7	Reference	
No and don’t know (n = 588)	461	78.4	1.31 (0.81 – 2.13)	0.261

$: US dollars; OR: odds ratio; CI: confidence interval; BMI: body mass index; PTLBW/FTLBW: preterm low birth-weight/full-term low birth-weight; ECC: early childhood caries.

## Discussion

The present study assessed the relationship between ECC and obesity among preschool children, controlling for covariates such as gender, diet, oral hygiene practices, socioeconomic factors, birth term, and birth weight. The overall prevalence of ECC was 74.3%, with high mean dmft (5.91) and dmfs (8.92) scores. The prevalence of ECC reported in the current study is close to the previous point-prevalence studies conducted in Saudi Arabia.^3,4^ However, it was higher compared to the global prevalence of ECC.^30^ It might be due to a high sugar diet and improper oral hygiene practices among the studied population. ECC prevalence was slightly higher among boys than girls, but the difference was not statistically significant.

The present study reports a positive association of ECC and obesity, with an odds ratio of 2.59-fold risk of ECC than normal-weight children. The result agrees with previous studies which showed a strong association between ECC and obesity.^5,10,12,31^ The reason may the sugar consumption, a common risk factor that increased the likelihood of both the conditions.^20,23,28^ The children who had high sugar consumption were at twice the risk of having ECC. Along with high sugar consumption, recent studies pointed out that low salivary flow among obese children contributed to high caries risk.^17,26^ However, the present study did not estimate the salivary flow rate, due to a lack of consent from parents.

The present study reported a positive association between high family income and ECC prevalence (OR 2.04). This may be due to greater consumption of a caries-promoting diet among children with a high family income. In contrast, previous studies showed increased prevalence and severity of ECC among low-income families due to the lack of access to oral health care.^13^

In agreement with a recent meta-analysis, the present study result showed PTLBW to be positively associated with ECC (OR 2.15). This may be due to increased enamel defects, as well as associated systemic problems that increased the risk of ECC among children born prematurely.^25^

In agreement with other authors,^7,29^ the current study found a positive association between ECC and toothbrushing frequency ≤ 1 per day (OR 5.63). These results emphasise the need to establish measures that promote adequate oral hygiene habits in preschool children.^18^

The cross-sectional study design limits the establishment of a causal association between ECC and obesity. The influence of recall bias in dietary history on study results could not be ruled out.

## Conclusion

The present study showed a positive association between ECC and obesity among preschool children. Along with obesity, ECC was positively associated with sugar consumption, high family income, poor oral hygiene practices and preterm low birth-weight. These observations demand an integrated approach in the management of ECC by addressing the common risk factors for ECC and obesity, thus emphasising the importance of both the oral and general health of preschool children.
